# Life style related to blood pressure and body weight in adolescence: Cross sectional data from the Young-HUNT study, Norway

**DOI:** 10.1186/1471-2458-8-111

**Published:** 2008-04-09

**Authors:** Magnus H Fasting, Tom IL Nilsen, Turid L Holmen, Torstein Vik

**Affiliations:** 1 Department of Public Health and General Practice Faculty of Medicine, Norwegian University of Science and Technology, N-7489 Trondheim, Norway; 2 Human Movements Science Programme, Faculty of Social Sciences and Technology Management, Norwegian University of Science and Technology, Trondheim, Norway; 3 HUNT Research Centre, Faculty of Medicine, Norwegian University of Science and Technology, Verdal, Norway

## Abstract

**Background:**

The associations between physical activity, unhealthy dietary habits and cigarette smoking and blood pressure, overweight and obesity are well established in adulthood. This is not the case for similar associations in adolescence. Thus, the purpose of this study is to examine how physical activity, smoking status and dietary habits were related to overweight, obesity and blood pressure in a population of Norwegian adolescents.

**Methods:**

Weight, height, systolic (SBP) and diastolic blood pressure (DBP) were measured, and body mass index (BMI) was calculated among 8408 adolescents who participated in a population based study in 1995–97 in the county of Nord-Trøndelag. Internationally accepted cut-off values were used to determine if the adolescents were overweight or obese. The adolescents also completed a detailed questionnaire including dietary habits, physical activity and smoking habits. We calculated adjusted mean blood pressures and odds ratios for being overweight or obese for different exposure categories of life style variables.

**Results:**

Low levels of physically activity were associated with increased odds of being overweight (odds ratio (OR), 1.4; 95% confidence interval (CI), 1.1–1.8 in girls and OR, 2.0; 95% CI, 1.6–2.5 in boys) or obese (girls: OR, 3.1; 95% CI, 1.6–6.0; boys: OR, 3.7; 95% CI, 2.1–6.4). In addition, the least physically active girls had a 1.5 mmHg higher mean DBP compared with the most active (p-trend <0.001), and among boys this difference was 1.0 mmHg (p-trend < 0.001). Smokers were more likely to be obese (OR, 1.6; 95% CI, 1.1–2.5 in girls and 1.4; 95% CI, 0.9–2.1 in boys) compared with non-smokers. Smokers also had lower mean SBP than non-smokers; however, this finding was restricted to smokers with the lowest smoking exposure. Associations between dietary habits and weight status largely disappeared after adjusting for weight losing behaviour.

**Conclusion:**

In this population of adolescents low levels of physical activity were associated with higher mean DBP and higher odds of overweight or obesity. Smoking was also associated with higher odds of overweight and obesity. The paradoxical associations between healthy dietary habits and overweight and obesity are most likely an effect of reverse causality.

## Background

High blood pressure, overweight and obesity in adulthood are well-known risk factors for cardiovascular disease. The development of these risk factors starts early in life and may be present already in childhood and adolescence [1-4].

Certain life style habits, including unhealthy dietary habits, cigarette smoking and inactivity are risk factors for cardiovascular disease that in part may be mediated or modulated through effects on blood pressure and body weight. These life style factors may also be established during childhood and adolescence, however, their associations with weight status and blood pressure in childhood and adolescence are less clear than in adulthood.

Low consumption of vegetables and fruits has been reported to be associated with increased blood pressure in adults [5], whereas in children and adolescents this was not supported in two reviews of the available evidence [6,7]. Physical inactivity is in itself an independent risk factor for cardiovascular disease in adulthood [8,9]. In addition, high physical activity is associated with reduced blood pressure [10] and lower body weight [11]. The associations between physical activity and blood pressure are not established in adolescence [12,13]. Irregularity of meals, such as breakfast skipping, is associated with overweight and obesity in childhood and adolescence [14], but the cause of this association remains unclear [15]. Finally, the effect of cigarette smoking on weight status and blood pressure is less well studied in adolescence than in adulthood where smoking is found to be associated with increased arterial stiffness [16] and dyslipidemia [17].

Thus, the purpose of this study was to examine how physical activity, smoking status and dietary habits were related to cardiovascular risk factors, such as elevated blood pressure, overweight and obesity in a population of Norwegian adolescents.

## Methods

### Study population

This population based cross sectional study comprises the youth part (Young-HUNT) of the second Nord-Trøndelag Health Study (HUNT II), carried out in 1995–1997. In Young-HUNT, all adolescents (13–19 years) in secondary or high school, living in the county of Nord-Trøndelag, Norway, were invited to participate. Among the 9917 adolescents who were invited to participate, 8950 (91%) accepted the invitation and completed a comprehensive self-administered questionnaire, including questions on life style and health. Moreover, 8408 (85%) of the adolescents chose to participate at a clinical examination that included measurements on height, weight and blood pressure by specially trained nurses.

Adolescents younger than 13 (N = 127) or older than 19 years (N = 397) were excluded. Furthermore, all adolescents with incomplete data on blood pressure, height and weight were excluded, leaving 7908 (80% of the 9917 invited) eligible for analysis. The total number of adolescents within this age group in Nord-Trøndelag was 10281 in 1995 [18], which means that our study population comprises 77% of the total population.

### Outcome measures

Blood pressure was measured with the adolescent sitting comfortably in a chair. First, the upper arm circumference was measured to the closest centimetre. If the circumference was less than 24 cm, a small cuff was used, if it was between 24 cm and 34 cm a medium cuff was used, and if it was 35 cm or more, a large cuff was used. The measurements were done using an automatic oscillometric, upon inflation technique (507 N monitor, Criticare System Inc.). The adolescents rested for two minutes before the first measurement, and the second and third measurements were done with one-minute intervals. Blood pressure was measured manually when an automatic recording could not be obtained. The mean value of the last two measurements was used in the analyses. If only one or two measurements were recorded, the single value (N = 7), or the mean of the two (N = 8) was used.

Height and weight were measured by trained nurses using internally standardized meter measures and weight scales. The subjects wore light clothes (T-shirts and trousers) without shoes. Height was read to the nearest centimetre (cm) and weight to the nearest kilogram (kg). Waist circumference was measured at the height of the umbilicus, using a tape measure to the nearest centimetre. Body mass index (BMI) was calculated as body weight (kg) divided by the squared value of height (m). To define overweight and obesity, we used cut-off values for adolescents proposed by Cole et al [19].

### Study variables

In the questionnaire, the adolescents were asked to indicate how often they consumed sweets and vegetables. Answer options were "more than once per day," "once per day," "every week, but not every day," "less than once per week" and "never." For the purpose of the present study, these responses were classified into three groups "once per day or more", "every week, but not every day" and "less than once per week." In addition the adolescents were asked to indicate whether they had breakfast "every day;" "4–6 days per week," "1–3 days per week" or "less than three days per week or never."

The survey also had a question about weight loosing behaviour. The adolescents were asked to indicate their answer on the following question: "Are you trying to loose weight?" The options were "No, I'm comfortable with my weight," "No, but I need to loose weight" and "Yes."

Physical exercise was recorded as "Not during the average school day: How many days a week do you play sports, or exercise to the point where you breath heavily an/or sweat?" Response alternatives were: "every day," "4–6 days a week," "2–3 days a week," "1 day a week," "not every week, but at least one day every two weeks," "not every 14 days, but at least once a months," "less than once a month" and "never" [20]. The answers were grouped into three levels: "4 days per week or more," "1–3 days per week" and "less than 1 day per week." These questions have been validated in a WHO survey [20].

Current smokers were defined as those who answered "yes, I smoke daily" or "yes, I smoke occasionally, but not daily" to the question: "do you smoke?" with five response options: "yes, I smoke daily," "yes, I smoke occasionally, but not daily," "no, I don't smoke, but I used to smoke daily," "no, I don't smoke, but I used to smoke occasionally" and "no, I don't smoke." Those who answered that they smoked daily were also asked to indicate the number of cigarettes they smoked every day, and how long they had been smokers. This information was used to calculate pack-years of smoking exposure among the daily smokers, and to categorize the daily smokers into three groups; group 1: those with less than one pack year, group 2: 1 – 2 pack years and group 3: smokers who had more than 2 pack years of smoking exposure. Adolescents in high school (3487) were also asked if they ever had tried marijuana.

### Ethics

All participants, and parents of adolescents under the age of 16, signed a written consent to take part in the study. The study was approved by the Regional Committee for Medical Research Ethics and the Norwegian Data Inspectorate Board.

### Statistical analysis

We computed mean diastolic and systolic blood pressure with 95% confidence interval (CI) within each category of the lifestyle factors, using a general linear model. In multivariable analysis, we adjusted for age and height, and in additional models we also evaluated potential confounding by BMI and smoking. In supplementary analysis, we assessed the independent effect of each lifestyle variable (i.e. smoking, diet (vegetable consumption) and physical activity) on blood pressure by mutual adjustment in the regression model.

Binary logistic regression was used to compute age-adjusted odds ratio (OR) with 95% CI for overweight or obesity associated with the different life style exposures. In additional multivariable analyses we adjusted for weight loosing behaviour, and we also examined the independent effect of each life style factor by mutual adjustment. Trend tests across exposure categories were calculated treating the categories as an ordinal variable in the regression model.

Given the sample size of boys (N = 3926) and girls (N = 3982), and assuming a prevalence of an adverse life style in the population to be 20% (the case for smoking), the present study has a power of 80 % to detect a difference in mean systolic blood pressure of approximately one percent (mean diff of 1.5 mm Hg for boys, and 1.2 mm Hg for girls; alpha of 0.05; Two-tailed). All data were analyzed using SPSS 14.0 for Windows (Copyright © SPSS Inc., 1989–2005).

## Results

Characteristics of the population are summarized in Table 1.

**Table 1:** Characteristics of the population

Characteristic	Girls	Boys
No. of participants	3982	3926
Mean age, years (SD)	15.9 (1.65)	15.9 (1.64)
Mean systolic blood pressure, mmHg (SD)	119.6 (10.60)	125.1 (12.73)
Mean diastolic blood pressure, mmHg (SD)	63.6 (7.63)	65.2 (8.37)
Mean weight, kg (SD)	58.9 (10.25)	64.8 (13.55)
Mean height, cm (SD)	165.5 (6.33)	174.3 (9.48)
Mean BMI, kg/m^2 ^(SD)	21.4 (3.30)	21.2 (3.25)
Mean waist circumference, cm (SD)	70.5 (8.00)	75.7 (8.77)
Percent overweight	15.7	15.8
Percent obese	2.8	2.9

Blood pressure increased with increasing degree of overweight, both among girls and boys (Table 2). Obese girls had a mean systolic blood pressure of 134.0 mm Hg (95% CI: 131.5–136.5) compared to 118.3 mm Hg (95% CI: 118.0 – 118.7) in normal weight girls (p-trend < 0.001), and obese boys had a mean systolic blood pressure of 139.4 mm Hg (95% CI: 137.0 – 141.8) compared with 123.7 mm Hg (95% CI: 123.3 – 124.1) in boys with normal weight (p-trend < 0.001).

**Table 2:** Weight status and systolic (SBP) and diastolic (DBP) blood pressure (mm Hg)

		SBP, mm Hg			DBP, mm Hg		
			
Variables	N	Mean	95%CI	P-trend	Mean	95% CI	P-trend
Females							
Normal weight	3355	118.3	118.0–118.7	--	63.2	62.9–63.5	--
Overweight	517	124.5	123.6–125.5	--	65.1	64.5–65.7	--
Obese	110	134.0	131.5–136.5	<0.001	70.1	68.7–71.5	<0.001
							
Males							
Normal weight	3307	123.7	123.3–124.1	--	64.7	64.4–65.0	--
Overweight	505	131.1	130.0–132.3	--	66.8	66.1–67.6	--
Obese	114	139.4	137.0–141.8	<0.001	71.0	69.1–72.8	<0.001

Six hundred and thirty one (15.8 %) girls were trying to lose weight and 971 (24.4 %) girls indicated that they needed to lose weight, but did not actively try. Among boys, 183 (4.7 %) reported that they were trying to lose weight, while 462 (11.8 %) indicated that they needed to lose weight, but did not actively try to lose weight.

There were smoking data on 7767 of the adolescents. Of these, 1586 (20 %) were classified as smokers. A higher proportion of girls (23 %) than boys (18 %) were smokers (p < 0.001). Among the 1586 smokers, we had detailed information on smoking exposure in 1518, and 766 (50.5 %) of these were occasional and 752 (49.5 %) were daily smokers. Among the daily smokers 434 (57.7 %) had less than one pack year, 188 (25 %) had between one and two, and 130 (17.3 %) daily smokers had more than two pack years of smoking exposure. Thus, only four percent (N = 318) of the total population had a smoking exposure of one pack year or more. Among adolescents in high school, 330 (9.4 %) had ever tried marijuana.

### Clustering of life style factors

To study clustering of adverse life style factors we used consumption of vegetables as a proxy for unhealthy diet, due to the observed inverse associations between consumption of sweets and breakfast frequency and high blood pressure or overweight. One unhealthy life style factor was present in 2615 (33.1 %) adolescents, whereas 1032 (13.1 %) had the combination of two and only 196 (2.5 %) adolescents had three unhealthy life style factors.

Among adolescents with one adverse life style factor, unhealthy diet was more common among boys (p < 0.001), whereas a higher proportion of girls were less physical active (p < 0.001) and were more often smokers (p < 0.001). Among those with two adverse factors, smoking combined with low levels of physical activity was reported by 317 girls (8.0 %) compared with 190 boys (4.8 %; p < 0.001), while physical activity combined with unhealthy diet was reported by 212 (5.4%) boys compared with 161 girls (4.0 %; p = 0.005).

### Blood pressure and life style

Both in girls (Table 3) and boys (Table 4) lower levels of physical activity were associated with higher diastolic blood pressure.

**Table 3:** Association between life style factors and systolic (SBP) and diastolic (DBP) blood pressure (mm Hg) in girls.

		SBP, mm Hg			DBP, mm Hg		
Variables	N	Mean	(95% CI)	P-trend	Mean	(95% CI)	P-trend

Regularity of breakfast*							
Every day	2539	119.7	119.3–120.1	--	63.6	63.3–63.8	--
4–6 days per week	543	119.1	118.2–119.9	--	63.6	63.0–64.3	--
1–3 days per week	454	119.6	118.6–120.5	--	63.8	63.1–64.5	--
Less than 1 day per week	403	119.8	118.8–120.8	0.95	64.1	63.4–64.8	0.18
							
Consumption of sweets*							
Once per day or more	371	117.8	116.7–118.8	--	63.2	62.5–64.0	--
Every week, but not every day	2797	119.7	119.3–120.0	--	63.6	63.3–63.8	--
Less than once per week	746	120.3	119.6–121.1	<0.001	64.2	63.6–64.7	0.02
							
Consumption of vegetables*							
Once per day or more	1531	119.4	118.9–120.0	--	63.5	63.1–63.9	--
Every week, but not every day	1825	119.7	119.2–120.2	--	63.5	63.2–63.9	--
Less than once per week	561	119.9	119.1–120.8	0.32	64.5	63.9–65.1	0.03
							
Physical activity*							
4 days or more per week	889	119.5	118.8–120.1	--	62.8	62.3–63.3	--
2–3 days per week	1710	119.5	119.0–120.0	--	63.5	63.2–63.9	--
1 day or less per week	1359	119.7	119.2–120.3	0.51	64.3	63.9–64.7	<0.001
							
Smoking status*							
Non smokers	3052	119.8	119.4–120.2	--	63.7	63.5–64.0	--
Smokers	884	118.8	118.1–119.5	0.01	63.3	62.8–63.8	0.16

*Adjusted for age and height

**Table 4:** Association between life style factors and systolic (SBP) and diastolic (DBP) blood pressure (mm Hg) in boys.

		SBP, mm Hg			DBP, mm Hg		
Variables	N	Mean	(95% CI)	P-trend	Mean	(95% CI)	P-trend

Regularity of breakfast*							
Every day	2840	125.2	124.8–125.6	--	65.0	64.7–65.2	--
4–6 days per week	404	125.7	124.6–126.8	--	65.8	65.0–66.5	--
1–3 days per week	322	124.8	123.6–126.1	--	65.8	65.0–66.7	--
Less than 1 day per week	273	125.1	123.7–126.5	0.78	66.0	65.0–66.9	0.005
							
Consumption of sweets*							
Once per day or more	536	123.3	122.3–124.2	--	64.6	63.9–65.2	--
Every week, but not every day	2595	125.3	124.8–125.7	--	65.2	64.9–65.5	--
Less than once per week	688	126.8	125.9–127.6	<0.001	65.5	65.0–66.1	0.03
							
Consumption of vegetables*							
Once per day or more	1315	125.0	124.4–125.6	--	65.1	64.6–65.5	--
Every week, but not every day	1700	125.3	124.8–125.9	--	65.2	64.8–65.6	--
Less than once per week	788	125.6	124.8–126.4	0.25	65.4	64.9–66.0	0.29
							
Physical activity*							
4 days or more per week	1275	124.6	124.0–125.3	--	64.6	64.2–65.0	--
2–3 days per week	1483	125.5	124.9–126.1	--	65.3	64.9–65.7	--
1 day or less per week	1129	125.3	124.6–126.0	0.14	65.6	65.2–66.1	<0.001
							
Smoking status*							
Non smokers	3129	125.4	125.0–125.8	--	65.3	65.0–65.6	--
Smokers	702	123.9	123.1–124.8	0.003	64.6	64.0–65.2	0.04

*Adjusted for age and height.

Smokers also had lower systolic blood pressure compared with non-smokers (Tables 3 and 4). A similar, but weaker association was also observed for diastolic blood pressure. However, this difference in mean blood pressures was only present between never smokers and occasional smokers or smokers with less than one pack year of smoking exposure, whereas mean blood pressure values did not differ between never smokers and daily smokers with one pack year or more of smoking exposure (data not shown).

We also analyzed the association between dietary habits and blood pressure, and found increasing systolic and diastolic blood pressure with decreasing consumption of sweets both for girls (Table 3) and for boys (Table 4). In addition, among boys less regularity of breakfast was associated with increasing diastolic blood pressure (Table 4), whereas among girls decreasing consumption of vegetables was associated with increasing diastolic blood pressure (Table 3).

When BMI was added as a covariate increasing breakfast frequency was associated with increasing systolic blood pressure, both in girls (p-trend = 0.036) and boys (p-trend = 0.048), whereas among boys, the association between increasing diastolic blood pressure and decreasing breakfast frequency persisted (p-trend = 0.026). When BMI was included, all associations with sweets except systolic blood pressure in boys, and all associations except diastolic blood pressure in girls disappeared (data not shown). Adjusting for marijuana exposure did not affect the blood pressure estimates.

Girls with three unhealthy life style factors had a mean diastolic blood pressure of 64.5 mm Hg (95% CI: 63.0 – 66.0) compared with 64.2 (95 % CI: 63.6 – 64.2) for those with two, and 63.3 mm Hg (95% CI: 62.9 – 63.6) among those with no unhealthy life style factors (p-trend = 0.003). A similar trend for boys was not present.

Multiple regression analyses suggested that age and height explained 3% of the variance in systolic- and 5% of diastolic blood pressure in girls. In boys, age and height explained 17% of the variance in systolic- and 12% of the variance in diastolic blood pressure. In a model not including BMI, sweets and smoking contributed statistically significant to both mean systolic- as well as diastolic blood pressure in both sexes, whereas physical activity contributed to mean diastolic blood pressure in both sexes. However, adjusted R-square was only marginally affected, (~ one percent) by each of these life style factors. Consumption of breakfast and vegetables did not have independent effects in this model, except for a statistically significant effect of breakfast in diastolic blood pressure in boys.

When BMI was included in the models, the explained variance increased to 28% of systolic- and to 14% of diastolic blood pressure in boys, and to 13% of systolic- and 7% of diastolic blood pressure in girls. The effects of current life style factors were essentially the same, with the exception that the association between blood pressure and frequency of sweet consumption now largely disappeared, and only persisted for systolic blood pressure in boys.

### Overweight, obesity and life style

Decreasing levels of physical activity were associated with increasing odds of being overweight or obese (Table 5). Mean waist circumference also increased with decreasing physical activity (Table 6).

**Table 5:** Odds ratio for overweight and obesity related to life style

	Girls				Boys			
		
	Overweight		Obesity		Overweight		Obesity	
				
Variables	OR	95% CI	OR	95% CI	OR	95% CI	OR	95% CI
Regularity of breakfast*								
Every day	1.0	(Ref.)	1.0	(Ref.)	1.0	(Ref.)	1.0	(Ref.)
4–6 days per week	1.2	(0.9–1.6)	1.3	(0.7–2.3)	1.3	(1.0–1.7)	1.2	(0.7–2.2)
1–3 days per week	1.8	(1.4–2.3)	2.6	(1.6–4.3)	1.6	(1.2–2.2)	1.8	(1.0–3.2)
Less than 1 day per week	1.5	(1.1–1.9)	2.4	(1.4–4.0)	1.6	(1.1–2.1)	1.8	(1.0–3.4)
								
Consumption of sweets*								
Once per day or more	1.0	(Ref.)	1.0	(Ref.)	1.0	(Ref.)	1.0	(Ref.)
Every week, but not every day	1.7	(1.1–2.4)	1.0	(0.5–2.0)	1.5	(1.1–2.0)	1.0	(0.6–1.9)
Less than once per week	3.2	(2.2–4.7)	2.7	(1.3–5.8)	2.3	(1.7–3.2)	2.1	(1.1–4.1)
								
Consumption of vegetables*								
Once per day or more	1.0	(Ref.)	1.0	(Ref.)	1.0	(Ref.)	1.0	(Ref.)
Every week, but not every day	0.9	(0.7–1.0)	0.6	(0.4–1.0)	1.0	(0.8–1.2)	0.8	(0.5–1.2)
Less than once per week	1.0	(0.7–1.3)	0.8	(0.4–1.3)	1.1	(0.8–1.3)	1.4	(0.9–2.3)
								
Physical activity*								
4 days or more per week	1.0	(Ref.)	1.0	(Ref.)	1.0	(Ref.)	1.0	(Ref.)
2–3 days per week	1.2	(0.9–1.5)	2.2	(1.1–4.3)	1.4	(1.1–1.7)	2.1	(1.2–3.7)
1 day or less per week	1.4	(1.1–1.8)	3.1	(1.6–6.0)	2.0	(1.6–2.5)	3.7	(2.1–6.4)
								
Smoking status*								
Non smokers	1.0	(Ref.)	1.0	(Ref.)	1.0	(Ref.)	1.0	(Ref.)
Smokers	1.1	(0.9–1.4)	1.6	(1.1–2.5)	1.2	(1.0–1.5)	1.4	(0.9–2.1)

*Adjusted for age.

**Table 6:** Waist circumference related to life style

	Females				Males			
		
Variables	Waist circumference, cm				Waist circumference, cm			
	N	Mean	95%CI	P-trend	N	Mean	95%CI	P-trend

Regularity of breakfast*								
Every day	2539	70.1	69.8–70.4	--	2840	75.5	75.2–75.8	--
4–6 days per week	543	70.8	70.1–71.4	--	404	76.4	75.6–77.2	--
1–3 days per week	454	71.3	70.6–72.0	--	322	76.0	75.1–76.9	--
Less than 1 day per week	403	71.7	70.9–72.4	<0.001	273	76.4	75.4–77.4	0.04
								
Consumption of sweets*								
Once per day or more	371	69.7	68.9–70.5	--	536	74.7	74.0–75.4	--
Every week, but not every day	2797	70.2	69.9–70.5	--	2595	75.6	75.3–75.9	--
Less than once per week	746	72.0	71.5–72.6	<0.001	688	77.0	76.4–77.6	<0.001
								
Consumption of vegetables*								
Once per day or more	1531	70.6	70.2–71.0	--	1315	75.7	75.3–76.2	--
Every week, but not every day	1825	70.3	70.0–70.7	--	1700	75.5	75.1–75.9	--
Less than once per week	561	70.9	70.3–71.6	0.71	788	76.2	75.6–76.8	0.29
								
Physical activity*								
4 days or more per week	889	70.2	69.6–70.7	--	1275	75.2	74.7–75.6	--
2–3 days per week	1710	70.4	70.0–70.7	--	1483	75.5	75.1–75.9	--
1 day or less per week	1359	70.9	70.4–71.3	0.03	1129	76.5	76.0–76.9	<0.001
								
Smoking status*								
Non smokers	3052	70.3	70.0–70.6	--	3129	75.5	75.2–75.8	--
Smokers	884	71.2	70.7–71.7	0.003	702	76.4	75.8–77.0	0.01

*Adjusted for age

Female smokers had higher odds of being obese compared to non-smokers. A similar, but weaker trend was present among boys (Table 5). Smokers also had higher mean waist circumference than never smokers (Table 6). These associations disappeared after adjusting for weight loosing behaviour.

The OR of being overweight or obese increased with decreasing regularity of breakfast, and with decreasing frequency of sweets consumption (Table 5). Consistently, waist circumference increased with decreasing frequency of breakfast and sweets consumption (Table 6). However, after adjusting for weight loosing behaviour, these associations were largely attenuated; the OR of being overweight was reduced for boys who had breakfast less than one times a week from 1.6 (95% CI: 1.1–2.1) to 1.1 (95% CI 0.7–1.6) compared with boys who had breakfast daily, and for girls, the OR was reduced from 1.5 (95% CI: 1.1–1.9) to 0.9 (95% CI: 0.6–1.2). There were no associations between consumption of vegetables and being overweight or obese.

In multiple logistic regression analyses the effects of each adverse life style factor had independent effects on overweight, although the odds ratio for overweight in adolescents who had breakfast less frequently, or who were smokers were slightly attenuated or even became borderline statistically insignificant (data not shown). However, the odds of being overweight for the least active adolescents were unchanged, and for adolescents who consumed sweets seldom, the odds were even strengthened (data not shown).

## Discussion

In this population of adolescents we found that low frequency of physical activity was associated with higher mean diastolic blood pressure, and with increased prevalence of overweight and obesity. Smokers were more likely to be overweight and obese, but they had lower blood pressure compared with non-smokers; however, in this youth population smoking exposure was of short duration. The finding that those who reported infrequent consumption of breakfast and sweets had a lower prevalence of overweight and obesity could to a large extent be explained by adolescents who wanted to lose weight, or who felt that they needed to lose weight.

The large sample size and the precision of the estimated associations make chance an unlikely explanation for the observed associations, and together with the proportions of exposed and unexposed individuals it ensures sufficient statistical power to detect very small differences.

The high participation rate and the independent administration of the questionnaires and the physical examination reduce the possibilities of selection or information bias. None the less, it can not be excluded that adolescents who are overweight or obese want to report more healthy dietary habits than they in fact have. It may also be considered a limitation that the questions used to assess dietary habits have not been validated, however, similar questions have been used in comparable studies [21,22]. Thus, information bias may have contributed to some of the apparently paradoxical findings on the relation between dietary habits and being overweight or obese.

The homogeneity of the study population makes confounding by ethnicity and socioeconomic status unlikely. However, the results of the multivariable analyses suggested that the association between consumption of vegetables and blood pressure may have been confounded by other life style factors, and similar confounding may have contributed to the associations between smoking and overweight, and between irregular breakfast and overweight.

The most consistent finding in this study was that high levels of physical activity were associated with lower diastolic blood pressure and reduced risks of being overweight or obese. The finding of a small reduction in diastolic blood pressure with increasing physical activity was independent of the adolescents' BMI, and of other life style factors, and is consistent with studies in adults [23,24] and with a study of adolescents who were hypertensive [13]. However, studies in adolescents have reported inconsistent results [25-27], and a recent meta analysis of intervention studies on physical activity and blood pressure in adolescents concluded that there was no significant effect of a short term intervention (8 – 36 weeks) in adolescents with normal blood pressure [12]. This may not be surprising, but may in fact be consistent with our finding that the life style factors evaluated in this study, including physical activity, only marginally contributed to the explained variance in blood pressure. Most probably, the variance in blood pressure at this age is mainly explained by genetic and early environmental factors (i.e. *in utero *or early infancy) [4]. However, we speculate that when adverse life style factors in adolescents persist into adulthood, their adverse effect on BP is likely to increase. Thus, our results may suggest that modifying life style habits in adolescence, emphasising the importance of regular physical activity could play a role in the prevention of later hypertension, even if the direct effect in this age group is small.

There was an association between low consumption of sweets and higher systolic and diastolic blood pressures, both among girls and boys. However, these relationships were significantly attenuated when BMI was included in the model. This fact, in addition to the similar relationship found between overweight and sweets consumption might indicate that this effect is mediated through BMI. We also found an association between less frequent breakfast consumption and increasing diastolic blood pressure in boys, and multivariable analyses suggested that this effect was independent of other life style factors. It may be reasonable to speculate that this effect could also be mediated via an effect on BMI; however, the association was weak, only among boys and only for diastolic blood pressure. Thus, the finding should be interpreted cautiously.

The somewhat paradoxical finding that smokers have lower blood pressure than non-smokers has also been reported by others [28-30]. The mechanism behind this finding is unclear. Charlton and While found in a prospective study that children with low blood pressure at age 10 were more likely to be smokers by age 16 years [31]. In our study, occasional smokers had lower blood pressures than never smokers while there were no differences between daily smokers with greater than one pack year smoking exposure and never smokers. This may suggest that the decreased blood pressure among smokers is a transient phenomenon, or as the study of Charlton and While suggests, that smokers have lower blood pressure before they start smoking. As duration of smoking increases this difference may level off. Since, only a very low proportion of adolescents in this study had smoked more than one pack year, we were not able to address this question further.

The finding that adolescents with low physical activity were more likely to be overweight or obese compared with adolescents who were most active is in agreement with several longitudinal studies [32,33], as well as with a large systematic review including 162 305 adolescents from 34 countries [34]. Moreover, the multivariable analyses suggested that this association was robust and independent of other life style factors.

The inverse associations between regularity of breakfast and overweight and obesity are well known, and several explanations have been postulated [35-38]. We have already discussed the possibility of information bias. Moreover, the associations between this and other dietary habits and overweight or obesity were weakened or even disappeared when we adjusted for weight losing behaviour, or for other life style variables. As indicated in a recent review [39], cross sectional studies on this topic may be subject to the presence of reverse causation. That is, overweight subjects are skipping breakfast in response to their weight; skipping breakfast is not causing overweight. Most likely the same mechanism may explain the finding that adolescents who seldom consumed sweets, were more likely to be overweight or obese.

In line with other studies [40], smoking was associated with increased risks of being overweight and obese, however, logistic regression analyses suggested that this association partly may be confounded by other life style factors.

It may be considered a positive finding that only 15% of the adolescents had two or more unhealthy life style factors. However, among girls, higher diastolic blood pressure was associated with increasing number of unhealthy life style habits, and this may suggest a dose-response effect which may be a further indication of a cause – effect relation between these adverse life style factors and risk for later hypertension.

## Conclusion

The robust and consistent finding of this study is that low levels of physical activity were associated with higher mean diastolic blood pressure and with higher odds of being overweight and obese. Smokers also had higher odds of being overweight and obese, and smokers in addition had lower blood pressure than non-smokers; however, the latter finding may be a transitory phenomenon. The paradoxical associations between healthy dietary habits and overweight and obesity are most likely an effect of reverse causality.

## Competing interests

The author(s) declare that they have no competing interests.

## Authors' contributions

MHF analysed the data and drafted the article. TILN advised on statistics and critically revised the manuscript. TLH participated in the questionnaire preparation, discussion on the study design and critically revised the manuscript. TV advised on statistics, participated in the discussion on the study design and critically revised the manuscript. All authors read and approved the final manuscript.

## Pre-publication history

The pre-publication history for this paper can be accessed here:


